# Colorectal cancer burden and trends in a South Asian cohort: experience from a regional tertiary care center in Sri Lanka

**DOI:** 10.1186/s13104-017-2869-1

**Published:** 2017-10-30

**Authors:** P. C. Chandrasinghe, D. S. Ediriweera, J. Hewavisenthi, S. K. Kumarage, F. R. Fernando, K. I. Deen

**Affiliations:** 1 Department of Surgery, Faculty of Medicine, University of Kelaniya, Kelaniya, Sri Lanka; 2 Centre for Health Informatics, Biostatistics and Epidemiology, Faculty of Medicine, University of Kelaniya, Kelaniya, Sri Lanka; 3 Department of Pathology, Faculty of Medicine, University of Kelaniya, Kelaniya, Sri Lanka

**Keywords:** Colorectal cancer, South Asia, Epidemiology, Cancer burden

## Abstract

**Objective:**

Colorectal cancer (CRC) burden is increasing in the south Asian region due to the changing socio-economic landscape and population demographics. There is a lack of robust high quality data from this region in order to evaluate the disease pattern and comparison. Using generalized linear models assuming Poisson distribution and model fitting, authors describe the variation in the landscape of CRC burden along time since 1997 at a regional tertiary care center in Sri Lanka.

**Results:**

Analyzing 679 patients, it is observed that both colon and rectal cancers have significantly increased over time (pre 2000—61, 2000 to 2004—178, 2005 to 2009—190, 2010 to 2014–250; P < 0.05). Majority of the cancers were left sided (82%) while 77% were rectosigmoid. Over 25% of all CRC were diagnosed in patients less than 50 years and the median age at diagnosis is < 62 years. Increasing trend is seen in the stage at presentation while 33% of the rectal cancers received neoadjuvant chemoradiation. Left sided preponderance, younger age at presentation and advanced stage at presentation was observed. CRC disease pattern in the South Asian population may vary from that observed in the western population which has implications on disease surveillance and treatment.

## Introduction

Colorectal cancer (CRC) has the fourth highest incidence amongst cancers in the world and is same for the south east Asian region [1]. Although CRC is thought to be a rare disease in the Asian region, an increasing trend in its incidence has been observed lately [2, 3]. Data from the Sri Lankan National Cancer Registry indicates CRC to have the fourth highest incidence amongst cancers in men and seventh overall highest incidence [4]. Increasing trend in the CRC incidence in this region may be attributed to the rapidly changing socio economic demographics although the influence of genetic and biological factors of the Asian population may also play a role [2]. However there is a scarcity of reported data from this region pertaining to CRC. Although a national cancer register is in place in Sri Lanka the demographic and tumour related data that is being published is not robust [5]. Currently Sri Lankan cancer data is classified as class D by the WHO’s GLOBOCAN network due to the lack of regional data [6]. High quality detailed regional epidemiological data plays a key role in planning and decision making for the delivery of equitable health care. The region considered for the study is Gampaha District, located in the western province of Sri Lanka. This is the second most populated district in the country and contains an equal blend of urban, suburban and rural communities [7]. Characteristics of colorectal cancer in this part of the world have not been well documented and may vary from the rest of the world with regard to demographics and anatomy. Perera et al. in 2008 reported the CRC burden in the same region for the first time. They reported that a majority of the cancers were left sided (80%) and 28% of the patients were younger than 50 years which revealed the pattern of cancer in this region may vary from the expected norms [8]. Current study is aimed at analyzing the trends in demographic, anatomical and histological features of CRC presented to tertiary care surgical referral unit over an 18 year period. The aim of the study is to analyse the disease characteristics of a representative cohort from a South Asian population over a period of time.

## Main text

### Methodology

Patients presenting with colorectal cancer to the University Surgical Unit at the North Colombo Teaching Hospital, Sri Lanka from 1997 to 2014 were included in the study. All patients were entered into a prospective database and followed up. Ethical clearance was obtained from the ethics review committee of the Faculty of Medicine, of University of Kelaniya to review, follow up data from this database. Records were retrieved from this database and patients were categorized into four groups based on time periods; pre 2000 (1997–1999), 2000–2004, 2005–2009 and 2010–2014. Sex, age, anatomical location of the tumour, American Joint Committee on Cancer (AJCC) staging, T stage and differentiation were analysed. Five separate anatomical regions were identified; distal rectum, proximal rectum, sigmoid colon, descending colon and right colon. A 6 cm distance from the dentate defined distal and proximal rectum and tumours proximal to the splenic flexure were considered as right colon. All tumours were staged according to the AJCC stage and the histopathological assessment was done according to a departmental protocol. Demographic and tumour related data were compared over time and amongst different age categories. Annual caseloads were assessed during the 18-year period to determine the trends in the caseload, sex and age at presentation. Generalized linear models assuming Poisson distribution was used to model the variation in age, sex and number of cases over time since 1997 for both colon and rectal cancers in general and separately for CRC of each AJCC stage. We considered the percentage of males and the mean age of the annual caseload to evaluate the variations in sex and age. These two variables (age and sex) along with annual caseload were considered as variables dependent over time for the model fitting. A P value of 0.05 was considered as significant. R Programming language version 3.3.1 was used to conduct the analysis.

### Results

**Fig. 1:**
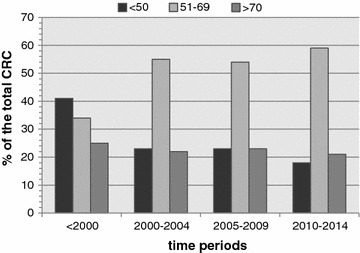
Percentages of CRC diagnosed for different age categories during each time period

A total of 679 patients with CRC presented over a 18 year period were studied (Male—346; median age—60 years; range 16–89). Mean follow up period of the population was 41 months. Figure 1 illustrates that the maximum contribution for the caseload is from those between 50 and 70 years. Table 1 depicts the sex, age categories, site and tumour stage (AJCC & T stages) for the population segregated to each time-period. Table 2 lays out the change in the caseload and patient demographics over time in relation to tumour site and stage, in a linear regression model.

**Table 1:** Demographic, anatomical and tumour related characteristics of the patient cohort

	Pre 2000	2000–2004	2005–2009	2010–2014
Sex				
Male	23	98	98	131
Female	38	80	92	119
Total (n = 679)	61	178	190	250
Age				
< 30	6	8	6	4
30–40	11	7	15	12
40–50	8	27	23	28
50–60	11	46	54	63
60–70	10	51	58	82
> 70	15	39	34	53
N/A	–	–	–	04
Rectum	33	121	123	168
Distal	14	54	45	87
Proximal	19	67	78	81
Colon	27	56	67	81
Sigmoid	3	4	7	22
Descending	12	26	22	21
Right	12	26	38	38
N/A	01	01	–	01
AJCC stage				
I	9	35	36	37
II	23	58	48	63
III	18	52	77	70
IV	06	23	14	09
N/A	05	10	18	71
T stage				
T1	2	13	14	15
T2	10	30	30	48
T3	33	94	85	94
T4	11	32	46	31
N/A	05	09	15	62

*N/A* not available

**Table 2:** Variation in the number of cases and patient demographics over time in relation to tumour site and stage

Parameter estimate from Poison regression model (P value)			
Model	Variables dependent over time		
	Number of cases	Mean age	Sex
Colon cancer	0.04 (< *0.01*)	0.12 (0.47)	0.01 (0.19)
Rectal cancer	0.05 (< *0.01*)	0.50 (*0.01*)	0.00 (0.31)
AJCC I	0.03 (*0.03*)	0.55 (0.07)	0.00 (0.88)
AJCC II	0.01 (0.15)	0.06 (0.74)	0.00 (0.89)
AJCC III	0.05 (*<* *0.01*)	0.20 (0.27)	0.00 (0.36)
AJCC IV	0.03 (*0.04*)	0.34 (0.38)	0.02 (*0.02*)

The significant P values are shown in italics

#### Anatomical distribution

Majority were rectal cancers (n = 445; 65%). Left sided cancers accounted for 82% of all CRCs while 77% were in the rectum or sigmoid region. Distal rectal cancers accounted for 45% of the rectal tumours. Five patients were diagnosed with FAP related cancers over the period. The numbers of both colon and rectal cancers diagnosed have steadily increased over time (Table 1). Poisson regression model confirms this increasing trend in the number of both colon and rectal cancers (P < 0.01) (Table 2).

#### Sex variation

There is no significant variation observed with regard to the sex of the patients in either colon or rectal cancers although majority has been males after 2000 (Tables 1, 2).

#### Age distribution

Median ages for the four time periods are 54, 60, 59 and 61 years respectively for all CRC patients although an increasing trend can be observed in the mean age for rectal cancer patients (Table 2). Of the total population 26.25% of the cancers are found in those less than 50 years. Except for the pre 2000 period highest percentage of CRC is seen in the 50–70 years age category and appears to be increasing (Fig. 1).

#### Tumour stage

There is an increase in the diagnosis of AJCC stage III and IV CRCs over time (Table 2). One hundred and forty seven (33%) of the 445 rectal cancers had received neoadjuvant treatment indicating a significant proportion has had locally advanced rectal cancers.

### Discussion

Colorectal cancer is traditionally considered to be a disease of the west although there is recent evidence to suggest a change in the epidemiological pattern due to fast changing socio economical parameters [9, 10]. Epidemiological data on CRC is lacking from the south Asian region and the available data is not of high quality. However the countries with a higher development index from the Asian region such as China, Japan and Korea has reported a two to four fold increase of CRC incidence [2]. South Asians who migrated to western countries have also reported high incidence of CRC probably owing to the change in lifestyle [11]. However the epidemiology of CRC appears to differ between the South Asian and other Asian ethnicities even in a western population indicating the presence of other biological factors responsible [12]. This article may not provide an exhaustive description of the burden of CRC in Sri Lanka, although the district in concern represents a well-balanced population cohort. A population based prevalence study with continuous centralized update of regional epidemiological data will be of value for health planning in this part of the world. Other authors have highlighted the need for mobilizing resources and increasing awareness of this developing epidemic in the Asian region [3]. Goh et al. reported a series of CRC diagnosed in a population of South East Asian patients who underwent colonoscopy for large bowel related symptoms [13]. In their series of 228 cancers 13% of the patients were below the age of 50 years and 68.5% of the tumours were in the recto-sigmoid region. In contrast the current study reports 26.25% of the cancers in less than 50 year olds, which is twice the amount as reported by Goh et al. This finding is consistent with the findings of Perera et al. studying the patients with CRC in the same district as this study in 2008 [8]. Mean age of the patients from their study was 64 years while in this cohort it is 58 years with median of 60 years. This may be an important finding with regard to the development of future guidelines for surveillance that may need to be at a lower age than recommended elsewhere. Also referring to Fig. 1; Table 1 it is apparent that the major burden of CRC is seen in the 50–70 years age group. The proportion of tumours found in the recto-sigmoid region is also higher in this population compared to the findings of Goh et al. (77% vs. 68.5%) although the total left sided tumours are in consistent with that of Perera et al. [8, 13]. This implies that in the presence of lower GI symptoms a flexible sigmoidoscopy as an outpatient patient procedure may have the potential to diagnose over 75% of the large bowel cancers in this population. This is important considering the scarcity of facilities in this region to carry out complete colonoscopy in large numbers, for disease surveillance. Majority of the patients presenting have had advanced T stage of the tumours in all time periods. Similar observation was made by Goh et al. in their study [13]. This phenomenon is seen in most countries in this region in the absence of screening guidelines. The increase in AJCC III over time is seen in this cohort, which means more tumours with nodal metastasis were diagnosed towards the latter part of the study period. This may be a true increase in advanced cancer due to changing tumour biology or an improvement in the pathological reporting of the specimens. Authors have previously shown that pro-forma based reporting of CRC specimens improved the standards of pathological assessment and the number of lymph nodes harvested has a positive effect on overall survival [14, 15]. Norwood et al. in 2009 studying a cohort of South Asian patients in the United Kingdom reported similar findings to this study [16]. Their patient cohort (n = 134) was younger, had more rectal cancers and the tumours were at an advanced stage at presentation compared to the Caucasian counterparts. Coupled with advanced stage, prevalence of more distal rectal cancers (45%) has resulted in 33% of the rectal cancers in the current study population requiring neoadjuvant treatment over the period.

In conclusion CRC in this cohort shows a steady increase over time. Majority of the tumours are in the rectum and are advanced at presentation. A significant proportion of the tumours are detected in those younger than 50 years and this amount is higher compared to other races. Findings of this study are in consistence with the few available reports from South Asian cohorts involving lesser number of patients. These variations if observed repeatedly in future studies from this region, may need to be envisaged in developing guidelines for primary surveillance of CRC in this region. These may require alterations to the existing guidance based on findings from the western populations especially with regard to the age at which screening is initiated. It is of importance to collect high quality regional data and carryout community based epidemiological studies to provide a clear picture of the CRC epidemiology in this region.

## Limitations

This study is based on a single center experience which could affect the actual pattern due to referral bias. However the authors wish to highlight the importance of accurate data collection and reporting from this region and the impact it has on decision making. Although the completeness of data is above 80% with regard to tumour stage, the missing data may have an effect on the final result. A multicenter study from the region would help to overcome the biases and provide more robust information regarding the case load and trend in colorectal cancer.

## Abbreviations

CRC: colorectal cancer
AJCC: American Joint Committee on Cancer
T stage: tumour stage
FAP: Familial adenomatous polyposis coli
PCC: Pramodh Chitral Chandrasinghe
DSE: Dileepa Senajith Ediriweera
JH: Janaki Hewavisenthi
SKK: Sumudu Kelum Kumarage
FRF: Filton Ranil Fernando
KID: Kemal Ismail Deen

## References

[1] Fact Sheets by Population. http://globocan.iarc.fr/Pages/fact_sheets_population.aspx.

[2] Sung JJ, Lau JY, Goh K, Leung W, Asia Pacific Working Group on Colorectal Cancer (2005). Increasing incidence of colorectal cancer in Asia: implications for screening. Lancet Oncol.

[3] Pourhoseingholi MA (2012). Increased burden of colorectal cancer in Asia. World J Gastrointest Oncol.

[4] Ministry of Health, Sri Lanka (2015). Cancer incidence data 2009.

[5] Ministry of Health, Sri Lanka (2010). Cancer incidence data 2001–2005.

[6] DataSource_and_methods. http://globocan.iarc.fr/Pages/DataSource_and_methods.aspx.

[7] Department of Census and Statistics of Sri Lanka website. http://www.statistics.gov.lk/PopHouSat/PDF/Population/p9p2%20Population%20by%20district%20,%20sex,%20sex%20ratio%20and%20population%20density.pdf.

[8] Perera T, Wijesuriya RE, Suraweera PHR, Wijewardene K, Kumarage SK, Ariyaratne MHJ (2008). Prevalence of colorectal cancer and survival in patients from the Gampaha District. North Colombo region. Ceylon Med J.

[9] Tamura K, Ishiguro S, Munakata A, Yoshida Y, Nakaji S, Sugawara K (1996). Annual changes in colorectal carcinoma incidence in Japan: analysis of survey data on incidence in Aomori Prefecture. Cancer.

[10] Yiu H-Y, Whittemore AS, Shibata A (2004). Increasing colorectal cancer incidence rates in Japan. Int J Cancer.

[11] Jain RV, Mills PK, Parikh-Patel A (2005). Cancer incidence in the south Asian population of California, 1988–2000. J Carcinog.

[12] Virk R, Gill S, Yoshida E, Radley S, Salh B (2010). Racial differences in the incidence of colorectal cancer. Can J Gastroenterol.

[13] Goh KL, Quek KF, Yeo GT, Hilmi IN, Lee CK, Hasnida N (2005). Colorectal cancer in Asians: a demographic and anatomic survey in Malaysian patients undergoing colonoscopy. Aliment Pharmacol Ther.

[14] Siriwardana PN, Pathmeswaran A, Hewavisenthi J, Deen KI (2009). Histopathology reporting in colorectal cancer: a proforma improves quality. Color Dis.

[15] Chandrasinghe PC, Ediriweera DS, Hewavisenthi J, Kumarage S, Deen KI (2014). The total number of lymph nodes harvested is associated with better survival in stages II and III colorectal cancer. Indian J Gastroenterol.

[16] Norwood MGA, Mann CD, Hemingway D, Miller AS (2009). Colorectal cancer: presentation and outcome in British South Asians. Colorectal Dis.

